# Unveiling the Interplay between the TLR4/MD2 Complex and HSP70 in the Human Cardiovascular System: A Computational Approach

**DOI:** 10.3390/ijms20133121

**Published:** 2019-06-26

**Authors:** Amanda Almeida de Oliveira, Josemar Faustino, Maria Elena de Lima, Ronaldo Menezes, Kenia Pedrosa Nunes

**Affiliations:** 1 Department of Biomedical and Chemical Engineering and Sciences, Florida Institute of Technology, Melbourne, FL 32901, USA; 2 Department of Computer Engineering and Sciences, Florida Institute of Technology, Melbourne, FL 32901, USA; 3 Grupo Santa Casa de Belo Horizonte, Programa de Pós-graduação em Ciências da Saúde, Biomedicina e Medicina, Ensino e Pesquisa da Santa Casa de Belo Horizonte, Belo Horizonte, MG 30150-240, Brazil; 4 Department of Computer Science, University of Exeter, Exeter EX4 4PY, UK

**Keywords:** TLR4/MD2 complex, HSP70, computational modelling, cardiovascular system

## Abstract

While precise mechanisms underlying cardiovascular diseases (CVDs) are still not fully understood, previous studies suggest that the innate immune system, through Toll-like receptor 4 (TLR4), plays a crucial part in the pathways leading to these diseases, mainly because of its interplay with endogenous molecules. The Heat-shock protein 70 family (HSP70-70kDa) is of particular interest in cardiovascular tissues as it may have dual effects when interacting with TLR4 pathways. Although the hypothesis of the HSP70 family members acting as TLR4 ligands is becoming widely accepted, to date no co-crystal structure of this complex is available and it is still unknown whether this process requires the co-adaptor MD2. In this study, we aimed at investigating the interplay between the TLR4/MD2 complex and HSP70 family members in the human cardiovascular system through transcriptomic data analysis and at proposing a putative interaction model between these proteins. We report compelling evidence of correlated expression levels between TLR4 and MD2 with HSP70 cognate family members, especially in heart tissue. In our molecular docking simulations, we found that HSP70 in the ATP-bound state presents a better docking score towards the TLR4/MD2 complex compared to the ADP-bound state (−22.60 vs. −10.29 kcal/mol, respectively). Additionally, we show via a proximity ligation assay for HSP70 and TLR4, that cells stimulated with ATP have higher formation of fluorescent spots and that MD2 might be required for the complexation of these proteins. The insights provided by our computational approach are potential scaffolds for future in vivo studies investigating the interplay between the TLR4/MD2 complex and HSP70 family members in the cardiovascular system.

## 1. Introduction

A prevalent worldwide condition, cardiovascular diseases (CVDs), take approximately 17.9 million lives annually [1]. While the precise molecular mechanisms underlying these diseases are still not fully understood, previous studies point to the innate immune system participating in the pathways leading to CVDs [2]. The link between innate immunity and CVDs has received much attention in recent years due to the possibility of developing therapeutic strategies to target the immune response. Particularly important in this context are the actions of the innate immune receptor Toll-like receptor 4 (TLR4), which recognizes exogenous and endogenous ligands [3]. Indeed, previous studies have highlighted TLR4 as a key player in the mechanisms of a number of diseases associated with the cardiovascular system including stroke [4], atherosclerosis [5], metabolic syndrome [6], hypertension [7], vasculogenic erectile dysfunction [8,9], and myocardium inflammation [10]. Activation of this receptor in cardiovascular tissues triggers a plethora of harmful effects that ultimately will lead to oxidative stress and low-grade inflammation [11,12,13]. Considering the functions allegedly played by TLR4, it is of little surprise that this receptor is the object of interest in a variety of studies, with many of them aiming at designing new pharmacological agents to target this pathway. However, targeting TLR4 itself for treating CVDs is challenging, mainly because of its primary role in innate immunity [14]. An alternative, in this case, would be to avoid its activation, which we could reach by characterizing ligands capable of modulating this receptor, especially those produced within the body. Although combined research efforts in the last two decades have elucidated an array of potential endogenous ligands for TLR4 [15], their interaction model is still a debatable issue.

The literature consistently shows an association between the Heat-shock protein 70 (HSP70 or HSPA-70kDa) family and TLR4 pathways [16,17,18,19]. In humans, 13 genes encode the proteins in the HSP70 family [20], many of which participate in protein folding and stabilization, basic mechanisms for maintaining proteomic homeostasis [21]. However, under chronic stress, members of this family have been suggested to interact with TLR4 triggering inflammatory responses [17,22], which might cascade severe implications in cardiovascular tissues. Although the association between HSP70 and TLR4 is well documented in different cell types [17,18,23], to date no co-crystal structure of this complex is available, and it is still unknown whether this process requires the co-adaptor MD2. Recent advances in the representation of the atomic structure of proteins through x-ray crystallography, nuclear magnetic resonance spectroscopy, and 3D electron microscopy combined with computational simulations have the potential to leverage our ability to understand the details of this interaction at the molecular scale. Additionally, there is no sizable human study investigating how the levels of different members of the HSP70 family correlate with TLR4 or MD2 at the transcriptome level.

**Figure 1:**
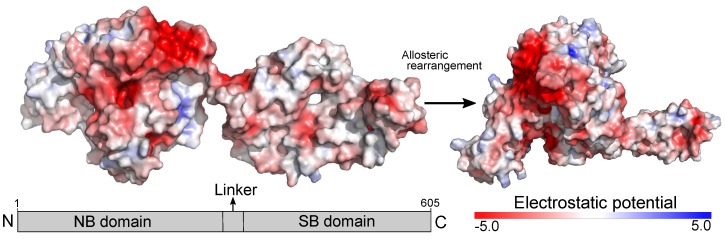
Overview of the allosteric rearrangement and electrostatic potential of HSP70. The protein structures (IDs 2KHO and 4B9Q) were retrieved from the Protein Data Bank and depicted to show the allosteric rearrangement [ADP (**left**) and ATP (**right**) bound states] and electrostatic potential of HSP70. The electrostatic potential was computed using the Adaptive Poisson-Boltzmann Solver (APBS) electrostatics plugin in the software PyMol (version 2.2.0) and it is shown in a red (negative) to blue (positive) scale.

The proteins within the HSP70 family are pervasive across all domains of life [24] having considerable sequence similarity among them [25,26]. Most of our understanding of the 3D structure and allosteric rearrangement of HSP70 comes from work conducted with the prokaryotic homologous DnaK [27,28]. Studies conducted with this protein showed that HSP70 has two conserved domains [nucleotide-binding domain (NBD) and (substrate-binding domain (SBD)] [29,30]. While the SBD binds client proteins, the NBD binds and hydrolyzes ATP. Hydrolysis of ATP regulates the thermodynamics and kinetics of the reactions where HSP70 is involved [27]. Likewise, the overall 3D structure of the protein undergoes extensively allosteric rearrangement upon hydrolysis of ATP, which affects substrate affinity [30,31]. HSP70 in its ADP state has a high affinity for substrates at the expense of reducing the rate of reactions, whereas HSP70 in its ATP state depletes substrate affinity and increases the speed of the reaction [27,29,30]. Besides changes in its 3D structure, the rearrangement cycle undergone by HSP70 affects the overall distribution of the electrostatic potential in the surface of the molecule. As shown in Figure 1, HSP70 has a negative surface potential, which might favor the interaction with positively charged receptors [32] such as the co-adaptor MD2 [33], a protein known for physically associating with TLR4 [34].

To fill in these gaps, in this study, we analyzed a large RNA sequence dataset from human cardiovascular tissues [heart (left ventricle) and aorta] aiming at understanding the interplay of each member of the HSP70 family with TLR4 and MD2 at the transcriptomic level. Additionally, we proposed a putative interaction mechanism between HSP70 (ADP and ATP bound states) and the TLR4/MD2 complex based on a series of rigid body molecular docking simulations and normal mode analysis, which was, at least in part, validated using a proximity ligation assay (PLA) for HSP70 and TLR4 in presence or absence of ATP and an inhibitor for MD2. Taken together, our findings provide valuable insights for future in vivo studies and might have a translational potential for designing new pharmacological agents within the cardiovascular system.

## 2. Results

### 2.1. TLR4/MD2 Complex Transcriptomic Levels Correlate with HSP70 in Human Cardiovascular Tissues

**Figure 2:**
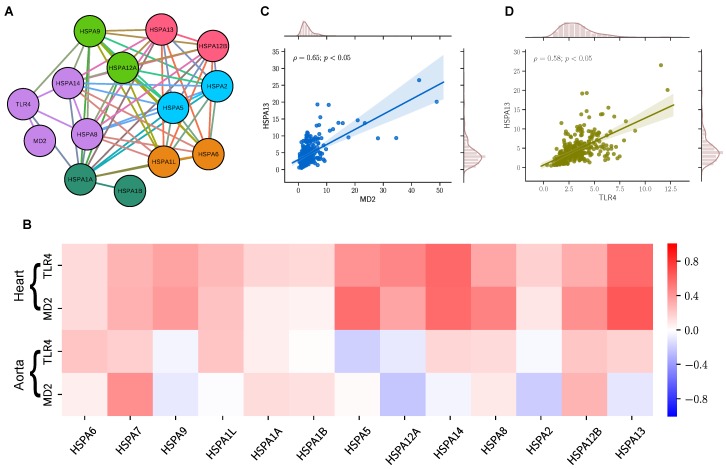
Evidence of protein-protein interaction in cardiovascular tissues. (**A**) Network interactivity of the genes of HSP70-family, TLR4 and MD2. Nodes are colored according to the community determined by the Louvain modularity algorithm. (**B**) Pearson ρ correlation matrix of HSP70-family with TLR4 and MD2 transcriptome expression levels of heart (left ventricle) and aortic human tissues plotted in a red (positive correlation), white (no correlation), and blue (negative correlation). (**C**,**D**) Scatter plots of the relationship between expression values in heart (left ventricle) of HSPA13 with MD2, and TLR4 respectively, as well as Pearson ρ correlation coefficient.

To unveil the interplay between the TLR4/MD2 complex and HSP70 family members, we constructed a protein-protein network using information from the STRING database [35]. The network contains 14 nodes (proteins), and 61 edges (connections) organized into six different communities (Figure 2A). The STRING database does not include information regarding HSPA7, which was long thought to be a pseudogene [25]. While TLR4 clustered with MD2, HSPA8, and HSPA14 suggesting a tight connection among them, it also interacted with HSPA9 and HSPA1A, an inducible protein [20]. We also observed a high clustering coefficient (0.912) and a small network diameter (3), which suggests an intertwined relationship between TLR4 and the HSP70 family members (Supplemental Table S1). Following our community detection analysis, we investigated whether this pattern of interaction would persist at the transcriptomic level in human cardiovascular tissues. We used a sizable RNA sequence dataset comprised of 602 samples derived from the heart (left ventricle; n = 303) and aorta (n = 299). Table 1 shows the donors’ history profile. The average donor was a male, in his 50s, and died after spending some time in a ventilator. In both tissues, heart (left ventricle) and aorta, we found some degree of correlation between the TLR4/MD2 complex and the members of the HSP70 family. However, this correlation was stronger in the heart (Figure 2B). The correlation values between TLR4 and MD2 in both tissues were used for comparison (Supplementary Figure S2). While the proteins that clustered with TLR4 in the network community analysis, HSPA8 and HSPA14, had lower correlation or were uncorrelated with TLR4 (ρ = 0.14 and ρ = 0.15, respectively) and MD2 (ρ = 0.09 and ρ = −0.04, respectively) in aortic tissues, they were positively correlated with TLR4 (ρ = 0.34 and ρ = 0.59, respectively) and MD2 (ρ = 0.49 and ρ = 0.58, respectively) in heart tissues. Likewise, the data revealed that HSPA13 levels, an HSP70 member expressed in the microsome [20] (vesicles that are present in unhealthy cells), positively correlate with the levels of MD2 and TLR4 in heart tissues (Figure 2C,D, respectively).

### 2.2. Putative Interaction Model between the TLR4/MD2 Complex and HSP70

**Figure 3:**
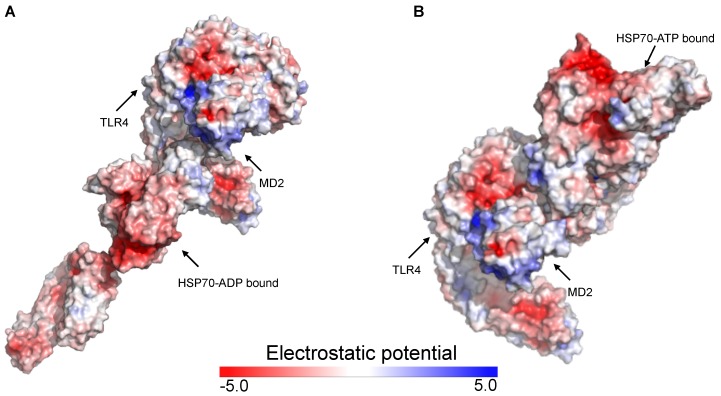
Electrostatic analysis of the interaction of the highest ranked docking conformation generated by ClusPro 2.0. (**A**) TLR4/MD2 vs. HSP70-ADP and (**B**) TLR4/MD2 vs. HSP70-ATP. The electrostatic potential of the highest ranked docking conformation for each condition was computed using the Adaptive Poisson-Boltzmann Solver (APBS) electrostatics plugin in the software PyMol (version 2.2.0) and it is shown in a red (negative) to blue (positive) scale.

Previous in silico studies that docked small molecules with MD2 or the TLR4/MD2 complex mainly considered the LPS-binding pocket as the possible ligand site. However, compared to LPS, HSP70 is a much larger molecule making its accommodation into the MD2 cavity difficult, which justifies the expansion of our search for the entire receptor surface. We first replicated the results of a previous study, which showed that the co-adaptor MD2 has a positive electrostatic potential whereas human TLR4 has a negative surface potential [33]. Because HSP70 has a negative potential, considering the electrostatic energy, MD2 could act as a gateway between HSP70 and TLR4. We conducted molecular docking simulations between HSP70 in its ADP (ID 2KHO) and ATP (ID 4B9Q) bound states with the human TLR4/MD2 (ID 3FXI) complex. We first analyzed how the electrostatic potential would affect the complexation of these molecules. Figure 3 demonstrates how the TLR4/MD2 complex might interact with HSP70 and the distribution of the electrostatic potential for the highest docked conformation of each condition.

**Figure 4:**
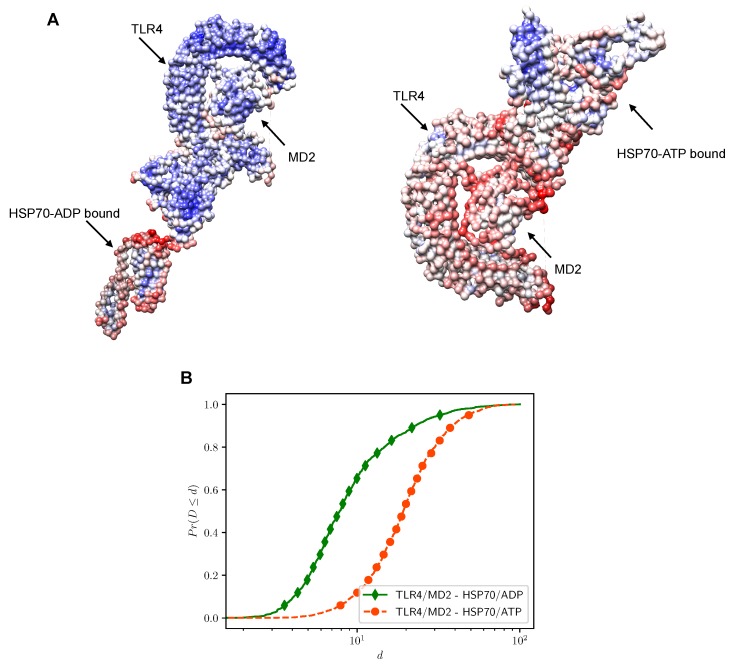
Normal mode analysis (NMA) of the TLR4/MD2 complex bound to HSP70 in the ADP (2KHO) or ATP (4B9Q) state. Structures were submitted to NMA in the web server iMODS and colored according to their estimated B-factor for deformability (**A**) using the software UCSF Chimera (version 1.12). The color scale ranges from blue (less flexible region) to red (more flexible region) in a logarithmic scale. The cumulative distribution function (**B**) shows the pattern of dispersion of deformability (*d*) throughout the docked conformations.

Next, we verified the interacting energy for the top 10 ranked conformations of each condition (Table 2). The interaction energy for the highest ranked conformation for HSP70 in the ADP bound state was higher (−10.29 kcal/mol) than the interaction energy for the ATP bound state (−22.60 kcal/mol). Although it is still unknown whether MD2 participates in the complexation of HSP70 and TLR4, our computational approach indicates that this reaction is energy favorable. Based on vibration analysis, we investigated how the interaction between ligand and receptor would affect the overall deformability of the system. The results for each conformation were compared to the conformational profile of each of the molecules alone, which we obtained before docking simulations. As previously discussed in other works, we observed that HSP70 in its ADP-state has a highly flexible region between the NBD and the SBD (linker region). On the other hand, the HSP70 ATP-bound had an overall structure more tightened together. Our results show that the complex formed by HSP70 in the ATP bound state and the TLR4/MD2 complex has a more flexible structure than the one composed by HSP70 in the ADP state (Figure 4). Although both simulated conditions were theoretically favorable, considering the interaction energy scores and binding sites deformability, the most favored docking situation occurred between the TLR4/MD2 complex and HSP70 in its ATP-bound state.

We next analyzed the formation of Hydrogen bonds between ligand and receptor as this interacting force can bring together two structures. Table 3 shows the interacting residues and the distance in *Å* of each Hydrogen bond between the TLR4/MD2 complex and HSP70 in the ADP and the ATP bound states. While in the ADP-bound state we identified 12 Hydrogen bonds, the ATP-bound state had 18, which could help explain why in this state the complex formed by HSP70 and the TLR4/MD2 complex has higher stability. Additionally, formation of Hydrogen bonds between HSP70 and MD2 were only observed in the ATP-bound state.

### 2.3. ATP Stimulates the Formation of Red Flurescence in PLA for HSP70 and TLR4

**Figure 5:**
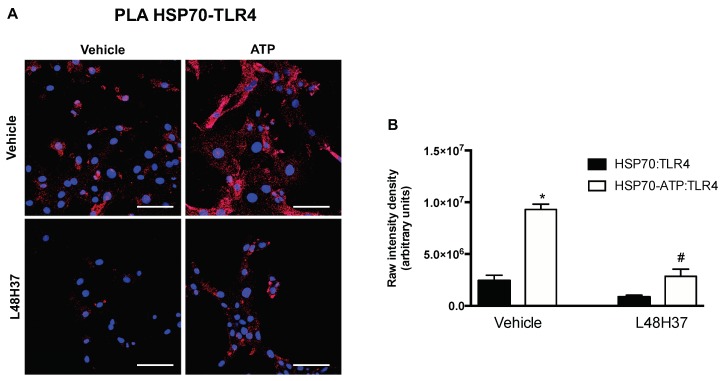
Proximity ligation assay produced red fluorescence spots, which suggests both target proteins (HSP70-TLR4) within interacting range. (**A**) Representative images (Scale bar: 20 μm). (**B**) The raw fluorescence intensity (red) was analyzed using the ImageJ software. *n* = 6 images per group. * *p* < 0.05 vs. HSP70:TLR4 vehicle treated and # *p* < 0.05 vs. HSP70-ATP:TLR4.

We conducted an in situ PLA to validate the overall patterns revealed by our computational approach. In this assay, formation of fluorescent spots indicates that both target proteins (HSP70-TLR4) are within interacting range. Primary cells were treated with rat recombinant HSP70 for 30 minutes in presence or absence of ATP and L48H37 (MD2 inhibitor). While fluorescence was also observed in non-treated cells, addition of ATP significantly increased the formation of fluorescent spots (Figure 5). Interestingly, MD2 blockade attenuated the number of fluorescent spots observed in these cells suggesting that the complexation of these proteins might require this co-adaptor.

## 3. Discussion

Diseases involving the cardiovascular system (heart and blood vessels) are the primary cause of death in the world [1]. While different components of immunity have been implicated in the pathways leading to these pathologies [3,36,37], the innate immune receptor TLR4 is thought to be one of the key contributors within the cardiovascular system [3,14,38]. Specifically because elevated levels of TLR4 were found in human atherosclerotic lesions [5]. Activation of this receptor increases nuclear translocation of the transcriptional factor nuclear factor (NF)-κB [39], which modifies the expression of pro-inflammatory cytokines such as Tumor necrosis factor (TNF)-α, stimulates mitogen-activated protein (MAP) kinases activity [40], and modulates the production of reactive oxygen species (ROS) [41]. Such scenario leads to severe implications in the patients’ clinical outcomes. Therefore, understanding the mechanisms involved in TLR4 activation, mainly those stimulated by endogenous ligands, is a focal point for unveiling potential targets within this pathway.

In this study, we used for the first time RNA sequence data from cardiovascular tissues [heart (left ventricle) and aorta] to show how the levels of different HSP70 family members, a long-known TLR4 endogenous ligand, correlate with this receptor and its co-adaptor MD2 in the human transcriptome. Previous studies have highlighted an array of endogenous ligands for TLR4, including HSP70 members [15]. More importantly, this protein family appears to participate in the mechanism of chronic diseases such as diabetes [42] and hypertension [43], which are two well-established risk factors for the development of CVDs. Still, the physiological mechanisms affected by presence of elevated HSP70 in cardiovascular tissues as well as how it is linked to impairment of the heart and blood vessels remains elusive. Because of the sequence similarity between human genes of HSP70 members, most of the studies investigating the actions of these molecular chaperones do not refer to specific proteins within the family. Likewise, results obtained with the bacterial homolog DnaK are often used when predicting behaviors of the human counterparts [27]. However, the implications related to the variations (TPM) of transcription expression levels of HSP70 family genes are still unknown. The human HSP70 family is encoded by 13 genes, which are expressed in the absence (cognate) or in the presence of stress (inducible) [20]. At this point, it is still less clear whether it is the cognate or the inducible HSP70s that act as ligands for TLR4, especially in cardiovascular tissues where limited data is available. Given the literature, one might expect that the inducible HSP70 genes (HSPA1A, HSPA1B, and HSPA6) would be the ones to be positively correlated with the TLR4/MD2 complex. However, we observed that they had lower correlation or were uncorrelated to the TLR4/MD2 complex (Figure 2). Interestingly, we observed that the cognate HSPA13 and HSPA14 genes are positively correlated with TLR4 and MD2 (Figure 2). Regarding HSPA13, to the best of our knowledge, besides the fact that this protein is expressed in the microsome and exerts housekeeping functions, the literature has limited information [20] leaving the nature of this relationship an open question. For HSPA14, in a previous study, Fang and collaborators have shown that TLR4 is essential for this protein to activate dendritic cells and, consequently, triggers inflammation and activation of MAP kinases [17], which could have serious implications in cardiovascular tissues. Considering that the HSP70 family plays ubiquitous roles depending on its cell localization, intracellular HSP70 is anti-inflammatory whereas extracellular HSP70 is pro-inflammatory, [22,23,44] it would be interesting to further investigate whether the increase in correlation, in this case, is due to a damage repair mechanism or if these proteins are acting as endogenous ligands for TLR4 or, even, if both conditions happen simultaneously. Regarding the other cognate genes, our results showed that their levels have low correlation in the heart or are uncorrelated in the aorta to both studied proteins. Nevertheless, further studies are needed to determine a causal relationship between the different members of the HSP70 family and the TLR4/MD2 complex.

The representation of the 3D structure for the human TLR4/MD2 complex ectodomain (ID 3FXI) [41] has opened new opportunities for researchers working in the field. Mainly, computational approaches such as the one we applied in this study have been used to uncover the details of the recognition mechanisms involving the TLR4/MD2 complex at the molecular level. While the literature has an extensive list of studies investigating how TLR4 interacts with its ligands or antagonists, there is limited knowledge regarding its complexation model with endogenous molecules, especially large proteins such as HSP70. To fill in this gap, we used rigid body docking simulation to propose a putative interaction model between the TLR4/MD2 complex and HSP70. Rigid body docking towards the TLR4/MD2 complex coupled with additional molecular simulations and experimental validation were previously conducted and successful results were obtained with large proteins such as S100A8 (ID 1MR8) [45] and HMGB1 (IDs 2RTU and 1HMF) [46]. The structural scaffold presented here provides insights into the complexation model between the molecular chaperone HSP70 and the TLR4/MD2 complex and may be used in further studies until a co-crystallized complex of these proteins is produced. While not entirely conclusive, our approach brings to light some properties of this complexation. The HSP70 family of proteins undergo extensive allosteric rearrangement using ATP as a thermodynamic and kinetic regulator (Figure 1), which affects their rate of reaction and substrate affinity [27,29,30]. Theoretically, it is possible for HSP70 in both the ADP and ATP states to interact with endogenous receptors such as the TLR4/MD2 complex. While HSP70 has a negative surface electrostatic potential, MD2 has a positive one [33], which might suggest a driving force in a potential interaction. In our model, the most negative electrostatic regions of HSP70 are not the ones used in the complexation with MD2 and TLR4 (Figure 3), we speculate that the geometry and size of the 3D structures of these molecules might be factors affecting this interaction pattern, still, further studies are needed.

In a previous study, aiming at elucidating the complexation model between HSP70 and the receptor for advanced glycation end product [RAGE (IDs 3CJJ and 4LP5)], the authors showed that the ADP bound state (ID 2KHO) also formed a complex with the receptor although it was less energetically favorable [32]. In our molecular docking simulations, we aimed at characterizing HSP70 affinity towards the human TLR4/MD2 (ID 3FXI) complex using both ADP (ID 2KHO) and ATP (ID 4B9Q) states. The utmost goal in molecular docking is to use algorithms to predict the likelihood that a given protein will bind to a target. Here, we identified the same pattern observed by Grunwald and collaborators [32] where the ATP-bound state of HSP70 formed a more stable interaction with the TLR4/MD2 complex (Table 2) with no significant deformability in the interface (Figure 4). This might be because the two domains of HSP70 in the ADP-bound state could rotate in opposite directions, making the protein a not very likely ligand, at least in this conformation, which was not observed in the ATP-bound state [32]. Of note, some regions of deformability (chain hinges) observed in the TLR4/MD2 complex vs. HSP70-ATP (Figure 4) were also present in our NMA before docking simulations. It was previously demonstrated that the flexibility of the helix connecting MD2 to TLR4 is essential for the ability of this co-adaptor to promptly close its hydrophobic pocket [47,48]. While high values of deformability are expected in flexible regions, low values are usually associated with rigid parts of proteins [49]. Still, it is important to consider that NMA predictions are based on the combination and interpolation of the modes generated for the studied protein dynamics, which occurs within the experimentally solved native state [50].

We found a larger number of Hydrogen bonds between the ATP-bound state and the TLR4/MD2 complex compared to the ADP-bound (Table 3), which could help to explain the underlying nature of this complex, especially because formation of non-covalent Hydrogen bonds might be evidence of stabilizing forces [51,52]. Interestingly, formation of Hydrogen bonds between HSP70 and MD2 is only observed when this protein is in the ATP bound state, which could be a potential mechanism contributing to the lower energy scores observed in this putative complexation model. The patterns generated in our computational simulations were next used as guidelines for the design of our in situ experimental approach, which was conducted aiming at validating our overall findings. The in situ PLA is an acceptable antibody method for detecting physical closeness of proteins [53]. Furthermore, this assay has been successfully used to study the interplay between HSP70 and RAGE [32] or NLRP3 inflamassome [54]. In this set of experiments, we exposed primary VSMCs to recombinant HSP70 in presence or absence of ATP and a small molecule inhibitor for the co-adaptor MD2. Corroborating the patterns revealed in our in silico simulations, addition of ATP led to higher formation of fluorescent spots in the in situ PLA, suggestive of both target proteins (HSP70 and TLR4) within interacting range. Additionally, presence of an inhibitor for MD2 attenuated the formation of fluorescent spots indicating that indeed this co-adaptor might play a role in the complexation of these proteins, at least, in cultured cells (Figure 5).

Altogether, the patterns identified in this work, whether with our RNA sequence data analysis, our computational simulations or our in situ PLA, provide insights that might be taken into consideration in future in vivo studies investigating this pathway within the cardiovascular system. The putative complexation model suggested in this work might have implications in future studies of the pathological cardiovascular conditions associated with high levels of TLR4/MD2 expression and the presence of HSP70 family members in the extracellular milieu. Unveiling the interplay between the TLR4/MD2 complex and HSP70 in the human cardiovascular system may help to bring to light new ideas for the development of novel CVDs’ therapeutic strategies.

## 4. Materials and Methods

### 4.1. Electrostatic Potential

The electrostatic potential of the studied proteins was calculated using the Adaptive Poisson-Boltzmann Solver (APBS) electrostatics plugin [55] in the software PyMol (version 2.2.0) [56]. The Protein Data Bank (PDB) files were prepared following the pdb2pqr method [57] and the results were visualized in a red (negative) to blue (positive) scale.

### 4.2. Network Evidence of HSP70 and TLR4 Protein-Protein Interaction

Protein-protein networks are a powerful methodological framework for studying the molecular interactions among proteins [58,59]. Using the STRING database [35] we generated a network with nodes representing the 13 proteins of the HSP70 family [20] plus TLR4 and MD2; and edges representing the relationships among them. The STRING database places an edge if an interaction is found based on: text mining, experiments, databases, co-expression, neighborhood, gene fusion, and co-occurrence. We disregarded the varied nature of these relationships and represented any interaction as an edge, which resulted in an undirected protein network (See edgelist supplementary file). Additionally, we applied a community detection method using Louvain modularity [60] to uncover a coarse-grained view of the network structure [61]. Network manipulation, community detection, and figure generation were performed using the software Gephi (version 0.9.2) [62].

### 4.3. RNA Sequence Data Analysis

Using the cmappy [63] and statsmodels [64] (version 0.9.0) Python packages, we computed the Pearson correlation (ρ) among the transcripts per million (TPM) values of RNA sequence expression of TLR4, MD2 and the HSP70 family genes occurring in the heart (left ventricle) and in the aorta of human donors. The correlation matrix visualization was generated using Python Matplotlib (version 3.0.0) [65]. A table with ρ and *p*-values for each case as well as distribution histograms is provided in the Supplementary Material (Supplemental Tables S2 and S3, and Figure S1, respectively). The RNA sequence data can be freely downloaded from the Genotype-Tissue Expression (GTEx) website at https://gtexportal.org/ (version 7, released on September 2017) [66].

### 4.4. Molecular Docking

The 3D structure of human TLR4 co-crystallized with MD2 [ID 3FXI, chain A (605 residues) and C (142 residues)] [41] in the presence of lipopolysaccharide (LPS) was retrieved from the Protein Data Bank to be used as the receptor. To avoid interference, we deleted the LPS molecules from the PDB file before docking simulations using the software UCSF Chimera (version 1.12) [67]. Because HSP70 is known to undergo allosteric rearrangement [28], we used both HSP70 in the ADP [ID 2KHO, chain A (605 residues)] [68] and ATP [ID 4B9Q, chain A (605 residues)] [69] states as ligands in the simulations. The computations were performed in the ClusPro 2.0 web server following its default guidelines for rigid body docking [70]. We conducted the simulations without restrictions, leaving the ligand free to search the entire TLR4/MD2 surface for the most favorable binding site. The top 10 ranked conformations generated in the ClusPro 2.0 *balanced* set of models, for each condition, were further analyzed. Using FoldX (version 4) [71] with GNU Parallel (version 20180922) [72] we repaired, optimized, and computed the interacting energy of each conformation model. The software UCSF Chimera (version 1.12) [67] was used for inspecting the conformation models for potential Hydrogen bonds.

### 4.5. Normal Mode Analysis

The highest ranked conformations for each condition were submitted for normal mode analysis (NMA) in the web server iMODS [49]. The results were attributed a common scale using Bioconda [73] and visualized in the software UCSF Chimera (version 1.12) [67], with the proteins colored according to NMA B-factors which represent the contribution of each residue to the overall deformability of the system. The residues are presented in a blue (less flexible regions) to red (more flexible regions) logarithmic scale. We also computed the cumulative distribution function of residues’ deformability using Python package statsmodels (version 0.9.0) [64] and plotted using Python Matplotlib (version 3.0.0) [65].

### 4.6. In Situ Proximity Ligation Assay

All animal procedures performed in this study are in accordance with the Guide for the Care and Use of Laboratory Animals from the National Institutes of Health and were reviewed and approved by the Institutional Animal Care and Use Committee of the Florida Institute of Technology (Protocol #2017/11). Vascular smooth muscle cells (VSMCs) were isolated from thoracic aorta of male Sprague Dawley rats (Taconic Biosciences, Rensselaer, NY, USA) with 4–6 weeks by explant method [74]. Briefly, aorta was harvested, cleaned, and cut into 1 mm^3^ piece. A total of 5 pieces were seeded into a 6-well plate in the presence of a small volume of Dulbecco’s Modified Eagle Medium (DMEM—an average of 0.5 mL) supplemented with 10% fetal bovine serum (FBS), 1% L-glutamine, and 1% penicillin/streptomycin. Plates were then placed in an incubator gassed with a mixture of 95% O${}_{2}$ and 5% CO${}_{2}$ at 37 °C. After 72 h of incubation, tissue was removed, and cells that migrated out of the tissue were maintained. VSMCs were characterized by its spindle morphology, typical growth pattern “hill and valley”, and positive immunoreactivity to α-smooth muscle actin 1st antibody: ab5694 diluted 1:100 (Abcam, Cambridge, UK) and, 2nd antibody: #35561 diluted 1:500 (Invitrogen, Carlsbad, CA, USA). After reaching 80% confluence, cells were treated with rat recombinant HSP70 (10 μg/mL) in the presence or absence of ATP (10 μM) and/or L48H37 (MD2 inhibitor; 10^−5^ M) for 30 min. Cells were then washed three times with 1x PBS and assayed using a PLA (DUO92101, Sigma-Aldrich, St. Louis, MO, USA) according to the manufacturer’s instructions. Plates were imaged (3 images per well) in a confocal microscope using a 20× objective. The raw fluorescence intensity was measured using the ImageJ software (NIH, Bethesda, MD, USA).

## Figures and Tables

**Table 1:** Genotype-Tissue Expression (GTEx) donor profile distribution by gender, age, and hardy scale death in cardiovascular tissues.

Variable		Heart (*n* = 303)	Aorta (*n* = 299)
Gender	Female	101	107
	Male	202	192
Age	20–29	21	26
	30–39	18	26
	40–49	50	51
	50–59	111	94
	60–69	96	97
	70–79	7	5
Hardy scale death	Fast	84	76
	Intermediate	13	15
	Slow	25	22
	Ventilator case	180	185

**Table 2:** Interacting energy (kcal/mol) and cluster size (n) of the top ranked docking conformations generated in the web server ClusPro 2.0.

Model	TLR4/MD2 vs. HSP70-ADP		TLR4/MD2 vs. HSP70-ATP	
	Energy (kcal/mol)	Cluster Size	Energy (kcal/mol)	Cluster Size
Model.000.00	−10.29	42	−22.60	47
Model.000.01	−20.29	36	−10.43	37
Model.000.02	−9.94	32	−20.23	33
Model.000.03	−7.86	25	−14.10	31
Model.000.04	−10.13	23	−17.91	27
Model.000.05	−8.72	21	−13.85	24
Model.000.06	−13.56	21	−26.70	24
Model.000.07	−11.00	20	−30.45	23
Model.000.08	−14.79	20	−15.02	22
Model.000.09	−20.92	18	−21.39	20

**Table 3:** Interacting residues and distance (*Å*) of the top ranked docking conformation generated in the web server ClusPro 2.0. Chain A represents HSP70 in the ADP (ID 2KHO) and ATP (ID 4B9Q) bound states and chain B represents the TLR4/MD2 complex (ID 3FXI).

TLR4/MD2 vs. HSP70-ADP			TLR4/MD2 vs. HSP70-ATP		
Donor	Aceptor	Distance	Donor	Aceptor	Distance
ARG56.A:NH1	GLU485.B:OE1	2.719	ARG56.A:NH1	GLU94.B:OE2	2.709
ASN61.A:N	GLU439.B:OE2	3.107	ARG56.A:NH2	GLU94.B:OE2	2.772
ARG261.A:NH1	GLN507.B:OE1	2.718	ARG261.A:NE	GLU89.B:OE2	2.868
TYR285.A:OH	GLN505.B:OE1	2.730	ARG261.A:NH2	GLU89.B:OE1	3.000
HIS431.B:ND1	ASP289.A:O	2.773	ARG261.A:NH2	GLU89.B:OE2	2.819
LYS435.B:NZ	ASP255.A:OD1	2.544	LYS268.A:NZ	GLN115.B:OE1	2.704
HIS458.B:ND1	ASP255.A:OD2	2.933	TYR285.A:OH	GLU42.B:OE1	2.762
ARG460.B:NH1	ARG56.A:O	2.609	ARG547.A:NE	GLU79.B:OE1	3.129
ARG460.B:NH2	LYS55.A:O	2.742	ARG547.A:NH2	GLU79.B:OE1	2.786
ARG460.B:NH2	GLN57.A:O	2.794	LYS577.A:NZ	SER76.B:OG	2.742
GLN484.B:NE2	ARG56.A:O	3.655	LYS20.B:NZ	GLN248.A:OE1	2.688
GLN484.B:NE2	GLN57.A:OE1	2.711	LYS20.B:NZ	ASP289.A:OD2	2.854
			TYR22.B:OH	THR291.A:OG1	2.647
			LYS47.B:NZ	GLN44.A:OE1	2.822
			ARG68.B:NH1	TYR285.A:OH	2.580
			ARG68.B:NH2	HIS295.A:NE2	2.969
			ARG87.B:NH2	ASN282.A:OD1	2.697
			SER100.B:OG	ASP540.A:OD1	2.860

## Supplementary Materials

The following are available online at https://www.mdpi.com/1422-0067/20/13/3121/s1, Table S1: Descriptive values for the protein-protein interaction network of TLR4/MD2 and HSP70 family, Table S2: ρ correlation values for TLR4, MD2 and HSP70 family genes, Table S3: *p*-values for the Pearson ρ correlation of TLR4, MD2 and HSP70 family genes, Figure S1: Distribution of RNA expression of TLR4, MD2 and HSP70 family genes, Figure S2: Scatter plots of the relationship between expression values in heart (left ventricle) (A) and aorta (B) of TLR4 with MD2, as well as Pearson correlation ρ coefficient, and a Supplementary edgelist file.
