# Supplementary material for: Unveiling the Interplay between the TLR4/MD2 Complex and HSP70 in the Human Cardiovascular System: A Computational Approach
Source: Int J Mol Sci. 2019 Jun 26;20(13):3121. doi: 10.3390/ijms20133121 (PMC6651210; doi:10.3390/ijms20133121)
Supplement: Supplementary file 1 [file ijms-20-03121-s001.zip › ijms-508448 sp for final/ijms-508448-supplementary.pdf]

Supplementary Materials: Unveiling the interplay between the TLR4/MD2 complex and HSP70 in the human cardiovascular system: a computational approach

Amanda Almeida de Oliveira[1,†], Josemar Faustino[2,†], Maria Elena de Lima[3], Ronaldo Menezes[4], and Kenia Nunes[1,*]

Description	Value
Number of nodes	14
Number of edges	61
Graph density	0.670
Network diameter	3
Average path length	1.417
Average degree	8.714
Number of communities	6
Average clustering coefficient	0.912

Table S1. Descriptive values for the protein-protein interaction network of TLR4/MD2 and HSP70 family.

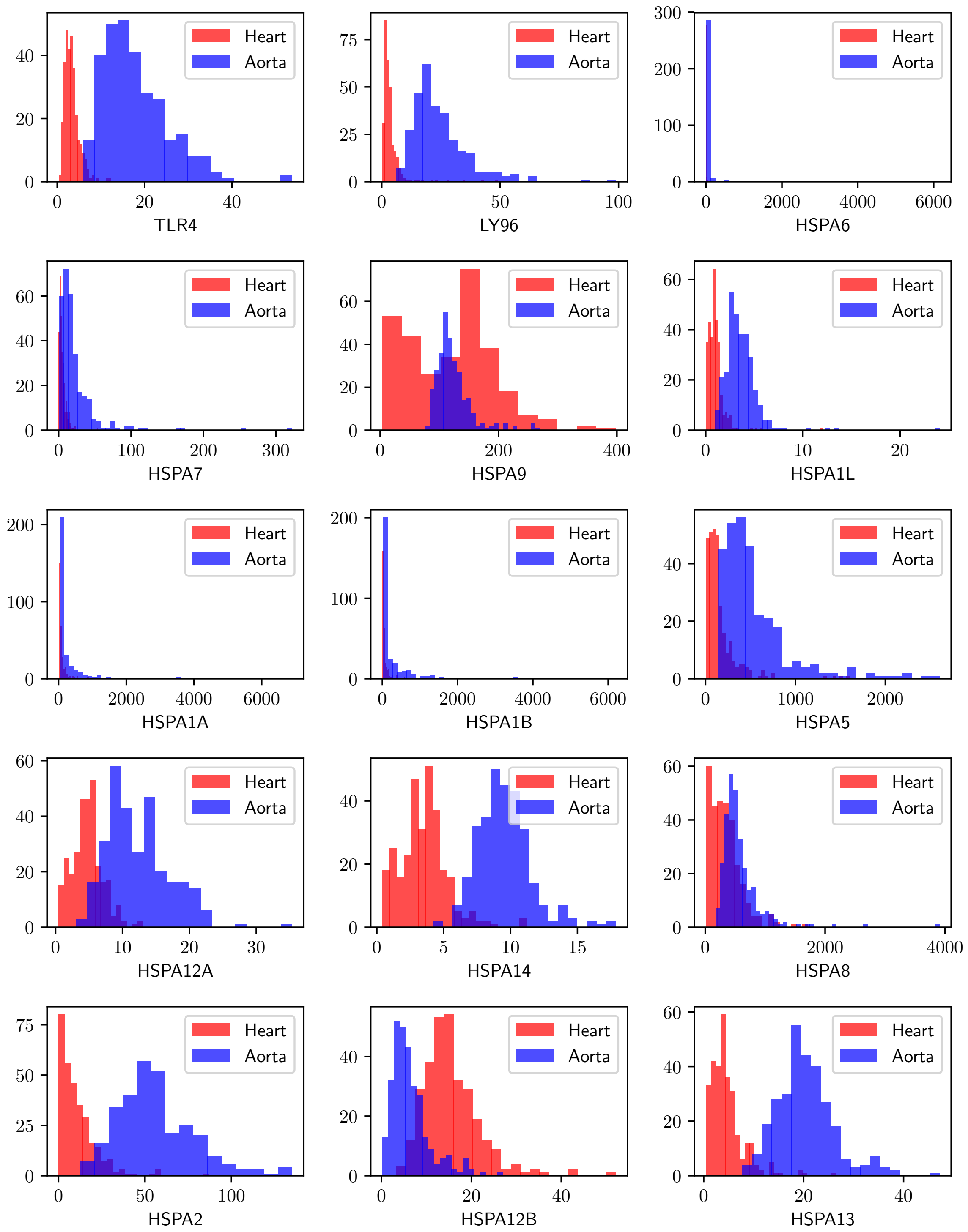

Figure S1. Distribution of RNA expression of TLR4, MD2 and HSP70 family genes.

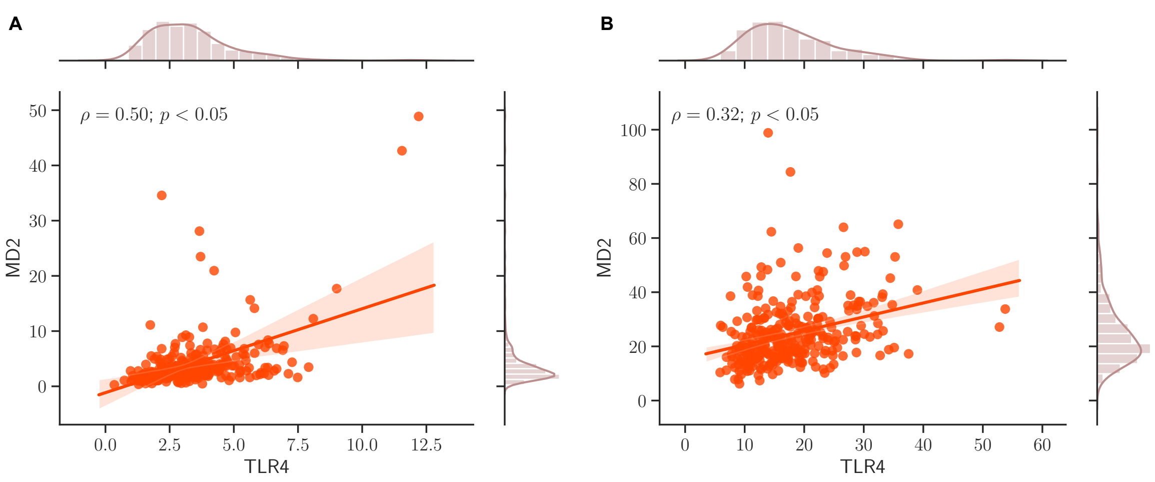

Figure S2. Scatter plots of the relationship between expression values in heart (left ventricle) (A) and aorta (B) of TLR4 with MD2, as well as Pearson correlation ρ coefficient.

		HSPA6	HSPA7	HSPA9	HSPA1L	HSPA1A	HSPA1B	HSPA5	HSPA12A	HSPA14	HSPA8	HSPA2	HSPA12B	HSPA13
Heart	TLR4	0.1470	0.2957	0.3664	0.2745	0.1696	0.1526	0.4282	0.4710	0.5911	0.3468	0.1855	0.3246	0.5841
	MD2	0.1411	0.3108	0.3932	0.2366	0.0681	0.0541	0.5690	0.3579	0.5809	0.4907	0.0997	0.4304	0.6523
Aorta	TLR4	0.2323	0.1935	-0.0413	0.2299	0.0641	0.0117	-0.1839	-0.0880	0.1598	0.1479	-0.0240	0.2230	0.1793
	MD2	0.0702	0.4447	-0.0897	-0.0101	0.1400	0.1214	0.0192	-0.2206	-0.0449	0.0937	-0.2006	0.2908	-0.0942

Table S2. ρ correlation values for TLR4, MD2 and HSP70 family genes

		HSPA6	HSPA7	HSPA9	HSPA1L	HSPA1A	HSPA1B	HSPA5	HSPA12A	HSPA14	HSPA8	HSPA2	HSPA12B	HSPA13
Heart	TLR4	0.0104	$\ll 0.01$	$\ll 0.01$	< 0.01	0.0031	0.0078	$\ll 0.01$	$\ll 0.01$	$\ll 0.01$	$\ll 0.01$	0.012	$\ll 0.01$	$\ll 0.01$
	MD2	0.0140	$\ll 0.01$	$\ll 0.01$	< 0.01	0.2370	0.3481	$\ll 0.01$	$\ll 0.01$	$\ll 0.01$	$\ll 0.01$	0.0833	$\ll 0.01$	$\ll 0.01$
Aorta	TLR4	< 0.01	$\ll 0.01$	$\ll 0.01$	0.0001	0.2695	0.8404	$\ll 0.01$	$\ll 0.01$	$\ll 0.01$	$\ll 0.01$	0.6800	$\ll 0.01$	$\ll 0.01$
	MD2	0.2264	$\ll 0.01$	$\ll 0.01$	0.8622	0.0154	0.0358	$\ll 0.01$	$\ll 0.01$	$\ll 0.01$	$\ll 0.01$	0.0005	$\ll 0.01$	$\ll 0.01$

Table S3. $p - values$ for the Pearson ρ correlation of TLR4, MD2 and HSP70 family genes
